# Vertebral fractures in a young woman: the impact of hypogonadism, glucocorticoids, and chronic disease

**DOI:** 10.1093/jbmr/zjag088

**Published:** 2026-05-23

**Authors:** Madhuni Herath, Hanh H Nguyen, Frances Milat, Peter R Ebeling

**Affiliations:** Centre of Endocrinology and Metabolism, Hudson Institute of Medical Research, Melbourne, VIC 3168, Australia; Department of Medicine, School of Clinical Sciences, Monash University, Melbourne, VIC 3168, Australia; Department of Endocrinology, Monash Health, Melbourne, VIC 3168, Australia; Department of Endocrinology and Diabetes, Western Health, Melbourne, VIC 3011, Australia; Department of Medicine, School of Clinical Sciences, Monash University, Melbourne, VIC 3168, Australia; Department of Endocrinology, Monash Health, Melbourne, VIC 3168, Australia; Department of Endocrinology and Diabetes, Western Health, Melbourne, VIC 3011, Australia; Centre of Endocrinology and Metabolism, Hudson Institute of Medical Research, Melbourne, VIC 3168, Australia; Department of Medicine, School of Clinical Sciences, Monash University, Melbourne, VIC 3168, Australia; Department of Endocrinology, Monash Health, Melbourne, VIC 3168, Australia; Department of Medicine, School of Clinical Sciences, Monash University, Melbourne, VIC 3168, Australia; Department of Endocrinology, Monash Health, Melbourne, VIC 3168, Australia

**Keywords:** Osteoporosis, vertebral fractures, glucocorticoids, hypogonadism, systemic lupus erythematosus

## Abstract

A premenopausal woman was reviewed for painful vertebral fractures in the context of prednisolone exposure for her newly diagnosed systemic lupus erythematosus (SLE). Known clinical risk factors for bone loss included exposure to depot medroxyprogesterone acetate (MPA) for 12 yr, cigarette smoking, a family history of osteoporosis and ongoing inflammatory arthritis. Initial investigations also identified vitamin D deficiency and low bone mass for age and sex. She was treated with vitamin D, and her contraception was changed to a levonorgestrel intrauterine device. In the context of further vertebral fractures confirmed with both MRI and bone scan and having had additional secondary causes of low bone mass excluded, she commenced targeted osteoporosis treatment. We discuss the complexities of managing bone fragility in young adults and the impact of depot MPA, inflammatory disease (SLE), and glucocorticoid-induced osteoporosis (GIO) on younger adults. In this woman, antiresorptive therapy with zoledronic acid was recommended; we also explore the existing evidence-base for antiresorptive and anabolic therapies in younger adults, with a particular focus on GIO.

## Case description

A 30-yr-old woman was reviewed for symptomatic, minimal trauma vertebral fractures at T5, T6, T8, and T10 occurring over the past 3 yr. She had been diagnosed with systemic lupus erythematosus (SLE) 24 mo prior, and had a history of mild intellectual disability, depression, and anxiety. Her medications at initial endocrine review included hydroxychloroquine, methotrexate, and depot medroxyprogesterone acetate (MPA). The MPA was initiated 12 yr before at the age of 18 yr for contraceptive purposes. She had been exposed to 3 mo of high-dose prednisolone (30 mg daily maximum) immediately following her SLE diagnosis. Her doses of methotrexate were increased, and rituximab was added in the context of worsening inflammatory markers and arthralgias. The initial fracture at T10 was identified when investigated for chronic back pain which gradually developed over time. Of note, there had been a fall onto her back from standing height. A timeline of relevant dates and events is presented in Figure S1.

An MRI (Figure S2) conducted as part of her initial assessment identified the acute severe T5 compression fracture but revealed no other bony pathology. BMD measured by DXA demonstrated low bone mass for age and sex at the left FN [areal BMD (aBMD) 0.67 g/cm^2^ (Z-score −2.1), TH aBMD 0.776 g/cm^2^ (Z-score −1.8), and L2-L4 aBMD 0.855 g/cm^2^ (Z-score −1.9)]. She weighed 60 kg and was 160 cm in height following her final vertebral fracture.

She was also severely vitamin D deficient (serum 25OHD 25 nmol/L; 10 ng/mL), had an elevated ESR 71 mm/h (normal <20) and while there was no evidence of monoclonal bands on protein electrophoresis, there was evidence of persistently elevated polyclonal IgG (1.90-1.95 g/L; normal 5.2-16.0 g/L), consistent with her autoimmune disease or a bacterial infection. She had normal renal function (serum creatinine 48 μmol/L (normal 45-85 μmol/L), estimated glomerular filtration rate of >90 mL/min/1.73 m^2^); thyroid function (thyroid stimulating hormone 2.81 mU/L (0.5-5.0 mU/L)), and PTH (2.2 pmol/L; normal 2.0-8.5 pmol/L) levels. She had normal calcium (2.43 mmol/L) and phosphate (1.67 mmol/L) levels and had normal urine calcium excretion (4.3 mmol/L/24 h (2.0-7.5)). Bone turnover markers were above the expected mean for a premenopausal woman at CTx 514 ng/L (general population normal range 150-800 ng/L) and P1NP 91 μg/L (general population normal range 15-70 μg/L). A screen for coeliac disease was negative (normal anti-gliadin IgG antibody levels and tissue transglutaminase IgA levels (<7 U/mL) with normal total IgA levels (2.6 g/L)). She had evidence of normocytic anemia with a hemoglobin of 112 g/L (120-160) and MCV of 81 fL (78-98).

She smoked 10 cigarettes/day but did not consume >2 standard drinks/day of alcohol. She went through menarche at 13 yr of age and had regular menstrual cycles until the insertion of an implanon implant. There was no history of parental hip fracture, but her mother had osteoporosis with a prior Colles’ fracture and her father had a history of rib fractures. She was nulliparous, and there were no features suggestive of an underlying monogenic cause of bone fragility on examination. The patient was found to have low dietary calcium intake, with <600 mg dietary calcium intake daily.

## Clinical problem

This young woman is at high imminent risk for fracture with new vertebral fractures. Long-term exposure to MPA and her underlying inflammatory arthritis, chronic severe vitamin D deficiency, prior high-dose glucocorticoid exposure are all likely to have contributed to her bone fragility. Accurate fracture risk calculation tools are unavailable for younger adults aged <40 yr and guidance for the management of low bone density in premenopausal women is less established than that for older adults with osteoporosis.

## Differential diagnosis and investigation

Differential diagnoses for this woman’s bone fragility include secondary osteoporosis due to other chronic disease, primary osteoporosis due to an underlying genetic disease, osteomalacia secondary to chronic vitamin D deficiency, and low peak bone mass. Table 1 includes secondary causes which would be important to exclude in this patient.

**Table 1:** Secondary causes of bone fragility.

**Disorders which contribute to bone fragility**
**Primary hyperparathyroidism**
**Cushing’s syndrome/disease**
**Acromegaly**
**Hyperthyroidism**
**Hypogonadism**
**Type 1 diabetes mellitus**
**Chronic kidney disease stage IV-V**
**Connective tissue disease such as rheumatoid arthritis, systemic lupus erythematosus**
**Inflammatory bowel disease**
**Liver cirrhosis**
**Immobility: Stroke with hemiplegia, cervical cord injury, cerebral palsy, polio, other neurological, and musculoskeletal disease contributing to immobility/disuse**
**Systemic mastocytosis**
**Idiopathic hypercalciuria**
**Monogenic disorders contributing to bone fragility**
**Osteogenesis imperfecta**
**Hypophosphatasia**
**Genetic causes of hypophosphatemic bone disease**
**Pathologic causes of fracture**
**Paget’s disease**
**Skeletal metastases from prostate or breast cancer**
**Medications**
**Glucocorticoids**
**Serotonin-noradrenaline receptor**
**Anti-epileptics: phenytoin**
**Aromatase inhibitors**
**Gonadotrophin-receptor analogues**
**Depot-medroxyprogesterone acetate**
**Acquired hypophosphatemia: iron infusions, tenofovir disoproxil fumarate**
**Calcineurin inhibitors**

Due to financial constraints, genetic testing and bone microarchitectural examination was not pursued. It is important to note, however, that in younger adults with recurrent fracture, both genetic testing and microarchitectural examination is likely to provide valuable insights.

## Diagnosis

This woman was diagnosed with multifactorial secondary osteoporosis due to MPA-induced bone loss, bone loss in the context of chronic inflammatory disease and glucocorticoid-induced osteoporosis (GIO). Her family history of fragility fractures, severe vitamin D deficiency, and smoking history are also important risk factors for bone fragility.

## Treatment and progression

Her MPA was ceased, and she agreed to have a levonorgestrel intrauterine device (Mirena) inserted. Vitamin D supplementation was commenced, and she was confirmed to be vitamin D replete 3 mo later. She was also counseled regarding adequate dietary calcium intake.

Following a discussion about her reproductive goals which revealed that she did not desire to have a family, and after explaining the potential long-term impact of bisphosphonate therapy in this setting, intravenous zoledronic acid treatment was recommended for secondary fracture prevention.

In considering goal-directed therapy with rapid optimization of bone density, first-line anabolic therapy would ideally be recommended for this patient.^1^ However, the choice of treatment in our patient was influenced also by local health policy and financial limitations. Anabolic therapy was not available as subsidized first-line treatment at the time of evaluation of this patient for treatment. If she did receive anabolic therapy, follow-on therapy with bisphosphonates would be preferred given her younger age and the challenges with consolidating denosumab to minimize rebound bone remodeling in the event of need for treatment intermission or transition.

## Discussion

Vertebral fracture risk is highest in the year following an initial vertebral fracture and greatest in those with multiple or severe vertebral fractures. In addition to reduced quality of life, vertebral fracture is associated with increased mortality, including in those aged <60 yr.^2^

### Depot MPA and bone health

Medroxyprogesterone acetate is a progestin-only injectable contraceptive. Its impact on aBMD is likely due to its ability to induce a hypoestrogenic state through suppression of gonadotrophins, which could be particularly detrimental when used in young women during the time of peak bone mass attainment.

Bone loss with MPA exposure occurs particularly with use beyond 2 yr. The reversibility of its effects and the impact of long-term exposure require further exploration although increased risk of fracture is found in past-users as well as current users.^3^ Greater than 3-fold risk of fracture has been found in women <30 yr of age exposed to MPA for >2.5 yr^4^ and increased fracture risk with OR >1.45 is reported in premenopausal women exposed to depot MPA for >4 yr.^5^

In view of the available data, albeit through observational studies, the risks and benefits of the contraceptive efficacy of MPA in avoiding unplanned pregnancies and the potential detrimental impact on bone needs evaluation, particularly, if its prolonged use is being considered in a woman with additional risk factors for bone loss.

### SLE and skeletal fragility

Men and women with SLE are at high risk of vertebral, hip, and other fragility fracture and typically have lower bone density compared with their peers.^6^ Disease-related changes inherent to SLE appear to contribute to bone loss, although the exact mechanisms are unclear and appear to differ from those of other inflammatory arthropathies, such as RA. Morphometric vertebral fractures are observed in up to 30% of patients with SLE^6^ and deterioration of trabecular and cortical bone microarchitecture is observed early in the disease process even in the absence of low bone mass.^6^

In addition to typical risk factors for bone loss, such as increasing age, postmenopausal status, low vitamin D, and Caucasian race, disease-related risk factors for such as higher organ damage (as measured by measured by the Systemic Lupus International Collaborating Clinics/American College of Rheumatology Damage Index SLICC/ACR-DI) with history of stroke and epilepsy, lupus nephritis, presence of lupus anticoagulant, higher cumulative glucocorticoid exposure, and older age at diagnosis are also associated with bone loss.^6^

### Glucocorticoid-induced osteoporosis

The most pronounced bone loss occurs in the first year following glucocorticoid commencement, with up to 20% bone loss observed. Increased fracture risk is noted as early as within 3 mo of commencement at doses as low as 2.5 mg/d.^7^ The greatest fracture risk is with exposure to ≥15 mg/d prednisolone-equivalent (2.5-fold increase in hip fracture risk)^8^ or a cumulative dose of ≥1 g (1.6-fold increase in hip fracture risk). Vertebral fractures are increased by 2.6-fold in those exposed to at least 7.5 mg/d or a cumulative dose of ≥1 g prednisolone equivalent, and by 3.8-fold for those exposed to 15 mg or greater.^8^

The impact of glucocorticoids on bone occurs over two-phases and is multi-faceted (Figure 1). In the shorter term, exposure to higher-dose glucocorticoids results in increased bone resorption while long-term exposure leads to more pronounced deficits in bone formation for the duration of glucocorticoid therapy. These effects are exerted through multiple direct and indirect actions affecting osteoblasts, osteocytes, and osteoclasts.

**Figure 1:**
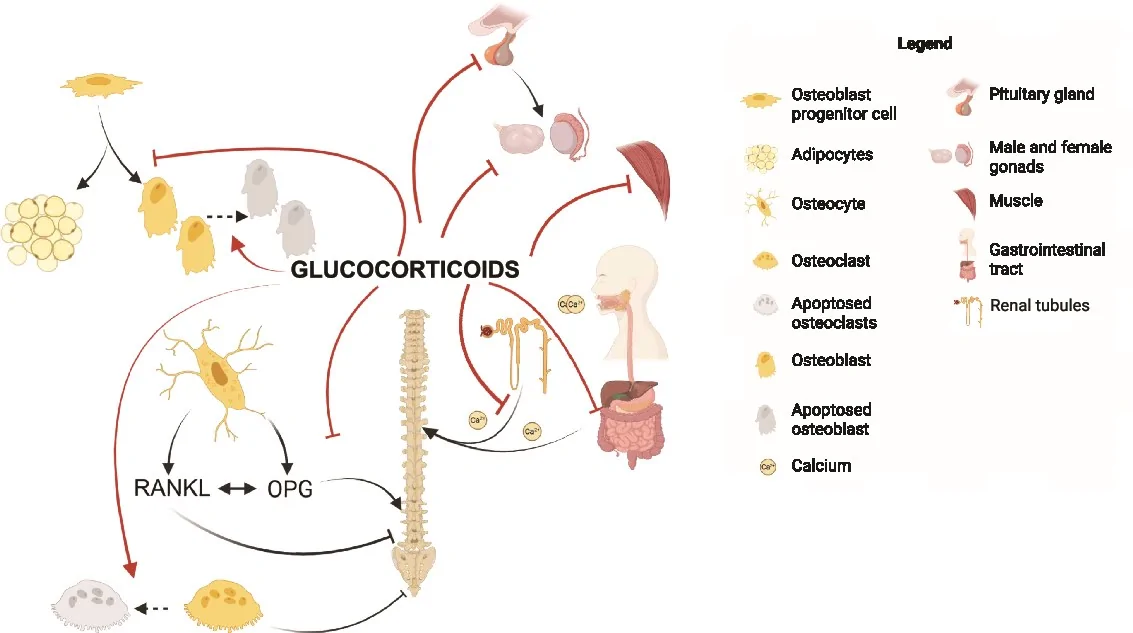
Glucocorticoids contribute to bone loss through direct and indirect actions on bone. Osteoblast differentiation is shifted toward pro-adipocytic lineage in the presence of glucocorticoids and osteoblast apoptosis is increased. The antiresorptive effect of osteoclasts are promoted through suppressive effect on osteoblasts and suppression of osteoprotegerin. Osteocyte function is inhibited and apoptosis is increased. Excess glucocorticoid exposure leads to increased renal calcium excretion through reduced renal tubular calcium reabsorption, reduced GI calcium absorption, and resultant secondary hyperparathyroidism; sarcopenia, hypogonadism, and reduced IGF-1mediated osteoblastic activity, all contributing to increased fracture risk.

Prolonged glucocorticoid exposure leads to muscle atrophy and weakness, leading to sarcopenia and further increasing falls and fracture risk. Intestinal calcium absorption is reduced and renal excretion of calcium is increased. Hypogonadism, through suppression of GnRH secretion and direct effect on the ovaries as testes, as well as reduced insulin-like growth factor 1 secretion and impairment of its activity in osteoblasts all contribute to reduced bone remodeling and growth with prolonged glucocorticoid exposure.^9^

Though exposure to high dose prednisolone was limited to 3 mo in this patient, the effect of chronic inflammation was likely to have also had a detrimental effect on bone.

### Treatment

Treatment of bone fragility in premenopausal women is complex. Unlike the management of postmenopausal osteoporosis, there are no tools for risk-stratification of fracture due to the low risk of fracture in the general population of younger adults and the multiple factors that can contribute to increased fracture risk in younger adults.^10^ International consensus for the management of bone fragility is lacking in the face of limited evidence; the recently published statement by ECTS-IOF provides a comprehensive overview and limited guidance.^11^

There is no international consensus regarding the treatment of GIO in premenopausal women and the regional recommendations vary considerably (Table S1), ranging from recommendation against the use of antiresorptive or anabolic therapy in premenopausal women with GIO, to considering referral to a bone specialist for those with a fragility fracture or low bone mass for age, to recommending bisphosphonate, parathyroid analogue, or denosumab for young adults with GIO. This variability highlights the paucity of good-quality evidence pertaining to younger adults, regional differences in availability of medications and the complexities of osteoporosis drug prescription particularly in the context of reproductive planning. Ethical considerations limit the availability of human studies. Neither anabolic agents, nor bisphosphonates, nor denosumab are safe for use in pregnancy; antenatal exposure to bisphosphonates, denosumab, or teriparatide may theoretically have long-term impacts on the fetal skeleton.^10^

There are only a few case reports or observational studies describing outcomes of antenatal bisphosphonate exposure: lower birth weight, transient hypocalcaemia, and spontaneous abortions have been described. Long-term follow-up is not available.^3^^,^^11^ Due to the long half-life of bisphosphonates, introduction of these agents in premenopausal women who have not yet completed their family requires a discussion of the potential and unknown risk to the skeleton of the offspring. Avoiding bisphosphonate exposure in a woman planning a pregnancy within 12 mo is recommended.^10^

Adequate vitamin D and calcium intake remain important in minimizing bone loss in premenopausal women with and without GIO. Oral and intravenous bisphosphonate therapy prevents bone loss in GIO,^3^ although data specific to SLE and in premenopausal women are less readily available. The use of denosumab in young, premenopausal women requires caution and careful consideration, particularly in the context of long-term use and increased risk of rebound bone resorption and multiple vertebral fractures on cessation. Estradiol therapy has been approached with caution in women with SLE due to the elevated thromboembolic risk in SLE.

Anabolic therapy has shown promise in treating GIO as well as premenopausal idiopathic osteoporosis. Teriparatide improves areal bone density in premenopausal women with idiopathic osteoporosis^12^ and is superior to alendronate in reducing vertebral fracture incidence and improving aBMD in adults with GIO.^13^ Sequential treatment following teriparatide is an important consideration for preservation of bone mass. Denosumab has been shown to further improve volumetric BMD in premenopausal women^14^; transition to zoledronic acid was noted to result in loss of aBMD by 2.4 ± 1.0% at the LS by 12 mo while alendronate was able to maintain aBMD for 12 mo at the LS, TH, and FN.^15^ Similar studies in premenopausal women with glucocorticoid exposure have not been replicated. Romosozumab has been shown to be beneficial in improving bone density in premenopausal women with GIO,^16^^,^^17^ anorexia nervosa,^18^ and its use has been described in a women with SLE and hypogonadism. Patients, particularly those with higher thromboembolic risk, such as in women with SLE, should be appropriately assessed and counseled regarding the adverse cardiovascular events reported with romosozumab exposure.

## Unanswered questions

There are many unanswered questions when considering the management of bone fragility in younger adults. The fracture risk, bone loss, and potential for recovery of BMD is unclear in young women exposed to >4 yr of MPA. The impact of SLE on fractures in young-adulthood and the mechanisms specific to SLE, which promote bone loss, warrants further investigation. Careful consideration of the impact of chronic disease and its treatment needs to be given, particularly in childhood or young adulthood, when peak bone mass is being attained. Further research into the best multi-modal approach for primary and secondary prevention of fracture risk in patients with SLE is essential.

## Conclusions and future directions

Young adults with fragility fracture require a comprehensive bone health assessment and treatment of identified risk factors. A framework including the development of absolute risk tools for identifying premenopausal women with highest risk of fracture would be valuable in streamlining investigations and targeting primary prevention measures, including anti-osteoporotic therapy. Such a framework would be an important step forward in improving quality of life and skeletal outcomes, particularly in younger adults with chronic inflammatory disease.

**Key Points**

- Bone loss is significantly increased in patients with SLE; glucocorticoid exposure further exacerbates this loss and increases fracture risk.
- Vertebral fractures are common in glucocorticoid-induced osteoporosis and occur at a higher aBMD than in postmenopausal osteoporosis.
- Vitamin D deficiency is common in patients with SLE and serum 25OHD levels should be checked and vitamin D supplementation initiated in those with deficiency.

## Supplementary Material

Supplementary_Materials_zjag088

## Data Availability

Data used for this paper are availble on request.
