# Supplementary material for: Vertebral fractures in a young woman: the impact of hypogonadism, glucocorticoids, and chronic disease
Source: J Bone Miner Res. 2026 May 23;41(11):1180–5. doi: 10.1093/jbmr/zjag088 (PMC13716917; doi:10.1093/jbmr/zjag088)
Supplement: Supplementary file 1 [file supplementary_materials_zjag088.zip › Supplementary Table 1_revised.docx]

Supplementary Table 1: Summary of International Guidelines for the Management of GIO

| Publishing Society/Group, Year | Stratification models | Recommendations |
| --- | --- | --- |
| American College of Rheumatology (ACR) 2022 | Moderate fracture risk: GC ≥7.5mg/day for ≥6 months AND BMD Z-score <-3 OR significant BMD loss  Very high risk: Prior fractures OR GC ≥30mg/day OR cumulative ≥5g/year | Treat adults <40 years at moderate or high risk of fracture with bisphosphonates, parathyroid analogues or denosumab; Recommendation against romosozumab for this group  Raloxifene may also be considered in solid transplant recipients other than renal transplant. Renal transplant patients to be referred for expert evaluation. |
| ECTS, 2022 | - | Commence vitamin D and calcium in all premenopausal women. Consider referral of all with a Z-score ≤-2, a history of fragility fracture and/or Genant ≥2 vertebral fracture to a bone specialist to determine whether anti-osteoporosis medication is indicated |
| Belgian Bone Club, 2022 | Premenopausal women aged >40 years   - Very high fracture risk: prior vertebral or hip fracture, T-Score ≤-2.5 - High fracture risk: increased FRAX^®^ score, T-Score ≤-1.5 or use of ≥7.5mg/day prednisolone equivalent | Recommends pharmacotherapy-based GIO prevention in premenopausal women (and men) aged >40 years with high/very high fracture risk with alendronate, risedronate, zoledronate, denosumab or teriparatide. Suggested to consider oral bisphosphonates in high risk and denosumab, teriparatide and zoledronate in very high-risk patients. In pre-menopausal women (and men) aged <40 years, prevention can be considered. |
| JSBMR, 2023 |  | Advises the use of lifestyle management in premenopausal women and suggests the use of bisphosphonates and PTH analogues as first and second-line treatment, respectively, in premenopausal women when treatment is necessary. The guidelines recommend against the use of bisphosphonates and PTH analogues in pregnant or breastfeeding women. |
| Thai Osteoporosis Foundation (TOPF), 2023 | Does not apply to premenopausal women (or men <50 years of age) | - |
